# Influence of the Cross-Link Density on the Rate of Crystallization of Poly(ε-Caprolactone)

**DOI:** 10.3390/polym10080902

**Published:** 2018-08-11

**Authors:** Igor Sedov, Timur Magsumov, Albert Abdullin, Egor Yarko, Timur Mukhametzyanov, Alexander Klimovitsky, Christoph Schick

**Affiliations:** 1 Chemical Institute, Kremlevskaya 18, Kazan Federal University, 420008 Kazan, Russia; timomax@mail.ru (T.Ma.); alb3978@yandex.ru (A.A.); yarkoeg@gmail.com (E.Y.); timmie.m@gmail.com (T.Mu.); aklimovi@mail.ru (A.K.); 2 Institute of Physics and Competence Centre CALOR, University of Rostock, Albert-Einstein-Str. 23-24, 18051 Rostock, Germany

**Keywords:** poly(ε-caprolactone), cross-linking, crystallization kinetics, fast scanning calorimetry, differential scanning calorimetry

## Abstract

Cross-linked poly(ε-caprolactone) (PCL) is a smart biocompatible polymer exhibiting two-way shape memory effect. PCL samples with different cross-link density were synthesized by heating the polymer with various amounts of radical initiator benzoyl peroxide (BPO). Non-isothermal crystallization kinetics was characterized by means of conventional differential scanning calorimetry (DSC) and fast scanning calorimetry (FSC). The latter technique was used to obtain the dependence of the degree of crystallinity on the preceding cooling rate by following the enthalpies of melting for each sample. It is shown that the cooling rate required to keep the cooled sample amorphous decreases with increasing cross-link density, i.e., crystallization process slows down monotonically. Covalent bonds between polymer chains impede the crystallization process. Consequently, FSC can be used as a rather quick and low sample consuming method to estimate the degree of cross-linking of PCL samples.

## 1. Introduction

Poly(ε-caprolactone) (PCL) is a biocompatible and biodegradable polymer with good mechanical properties. It is produced by the polymerization of ε-caprolactone with an annual global production of tens of thousands of tons [1]. The low melting point (around 60 °C) of PCL makes it a convenient material for 3D printing and rapid prototyping. Prospective biomedical applications of PCL include the manufacturing of implants, especially scaffolds for tissue, bone, and cartilage engineering, surgical sutures, and other medical devices [2]. It is also possible to use PCL for the encapsulation of pharmaceuticals in micro- and nanospheres, which can be administered through ingestion or injection [3,4]. The rate of biodegradation of PCL in the human organism is lower than that of some other biocompatible polyesters, such as polylactic and polyglycolic acids [2,5], making it more applicable for long-term drug delivery systems.

The properties of PCL can be tailored by fabrication of PCL-based blends and composites, either by copolymerization of caprolactone, with different amounts of polyhydric alcohols or hydroxy acids. In addition, PCL is capable of cross-linking when treated with radical initiators. Cross-linked PCL has a greater mechanical strength, does not flow, even at temperatures above its melting point, and has a slower biodegradation rate [6], which may be useful to reduce the rate of drug release from encapsulated forms. A very interesting property of cross-linked PCL is its two-way shape memory effect [7]. The polymer can remember one shape at a low temperature and another shape at a high temperature and recover them after deformation for both heating and cooling, which is considered a step towards the development of programmable matter.

PCL has a low glass transition temperature (around −60 °C) and stays in a semi-crystalline rubbery state at room temperature. The degree of crystallinity of the polymer also affects its mechanical and other physical properties, as well as the rate of biodegradation [8,9]. In order to control crystallinity, an understanding of crystallization kinetics is important. The kinetics of crystallization of PCL and some of its blends [10,11,12,13] have been studied previously using differential scanning calorimetry (DSC), employing samples with masses of a few milligrams. However, during the processing of polymers, they are cooled at high rates and their crystallization occurs at a high supercooling. Conventional DSC is limited to hundreds K·min^−1^ cooling rates, which makes it impossible to study fast crystallization processes at low temperatures. In the last decades, fast scanning calorimetry (FSC) using chip sensors was developed, allowing heating and cooling of the polymer samples with rates up to millions K·min^−1^ [14,15,16,17,18,19,20]. Such controlled high cooling rates became available due to a dramatic reduction of the calorimeter and the sample size. Chip sensors use free standing silicon nitride membranes with addenda heat capacities down to 100 pJ∙K^−1^ at room temperature and comparable small sample heat capacities [14,18,21]. FSC is able to determine the rate of crystallization, as well as nucleation process, over a broad temperature range even for rapidly crystallizing polymers [22,23,24,25]. It was previously applied to study crystallization of pure PCL with various molecular masses [26,27] and some of its composites [28]. However, no FSC data on crystallization kinetics of cross-linked PCL have been reported up to now. Moreover, applications of FSC to study cross-linked polymers have yet to be done.

Studies of the influence of the degree of cross-linking on the parameters of polymer phase transitions can be useful for technological purposes as well as for the development of theoretical descriptions of phase transitions. There is also an interest in quick, robust, and low sample consuming methods to determine the cross-link density of polymer samples [29]. Thus, any physical property of the polymer that shows a dependence on its cross-link density is of potential use. In the present work, we study the crystallization behavior of PCL samples cross-linked using different amounts of benzoyl peroxide (BPO) by means of both conventional DSC and FSC. Our goal was to analyze the influence of the spatial density of cross-links between linear PCL chains on the characteristic features of the crystallization process.

## 2. Materials and Methods

### 2.1. Synthesis of Cross-Linked PCL

In order to prepare the samples of cross-linked PCL, commercial PCL (Aldrich, St. Louis, MO, USA, average *M*_n_ = 45,000 g·mol^−1^, density *ρ*_p_ = 1.142 g·cm^−3^) and benzoyl peroxide (BPO, Aldrich, 75%, remainder is water as a stabilizer) were taken in different proportions and dissolved in dichloromethane (Komponent-Reaktiv, Moscow, Russia, 99.85%). The solvent was evaporated, and the mixture was heated up to 150 °C and kept for 60 min at this temperature to cross-link the sample.

### 2.2. Equilibrium Swelling Experiments

The degree of cross-linking of the obtained samples was measured using an equilibrium swelling method. About 0.2 g of each sample were first swollen in boiling toluene (Komponent-Reaktiv, 99.85%) using a Soxhlet extractor (Medsteklo, Klin, Russia) for 8 h and then left to equilibrate with toluene at 25 °C for 48 h. The mass *m* of the swollen PCL was determined, then the specimen was dried in vacuum and weighed again to record the mass of the dry polymer *m*_p_. For each BPO:PCL ratio, the experiment was repeated four times with new cross-linked samples averaging the volume swelling ratio:

(1) $$Q=\frac{m_{p}/\rho_{p}+\left(m-m_{p}\right)/\rho_{s}}{m_{p}/\rho_{p}},$$

where *ρ_s_* = 0.862 g·cm^−3^ and *ρ_p_* = 1.142 g·cm^−3^ are the densities of toluene and the polymer, respectively. Repetitive swelling experiments with the same sample do not significantly change the values of *Q*. The spatial density of cross-links was calculated according to the Flory-Rehner equation [30]:

(2) $$N=\frac{-(\ln(1-v_{p})+v_{p}+\chi v_{p}^{2})}{V_{s}(v_{p}^{1/3}-\frac{v_{p}}{2})},$$

where *v*_p_ = 1/*Q* is the volume fraction of PCL in swollen state, $V_{s}$ = 1.06 × 10^–4^ m^3^·mol^−1^ is the molar volume of the solvent (toluene), and *χ* is the Flory-Huggins interaction parameter. The value of *χ* can be estimated according to the equation:

(3) $$\chi =\frac{{\left(\delta_{s}-\delta_{p}\right)}^{2}V_{s}}{RT}.$$

Using the literature values of Hildebrand solubility parameters for PCL at 25 °C *δ*_p_ = 19.7 MPa^1/2^ [31] and toluene *δ*_s_ = 18.2 MPa^1/2^ [32] yields *χ* = 0.10.

### 2.3. Conventional DSC Experiments

The DSC curves of PCL and the cross-linked samples were recorded with a Mettler Toledo DSC823 (Mettler Toledo, Greifensee, Switzerland) and a Perkin Elmer Pyris 1 DSC instruments (PerkinElmer, Waltham, MA, USA) at 5–200 K·min^−1^ scanning rates. Using these curves, the temperatures and enthalpies of phase transitions were determined, and the kinetics of the non-isothermal crystallization process were characterized.

### 2.4. FSC Experiments

**Figure 1:**
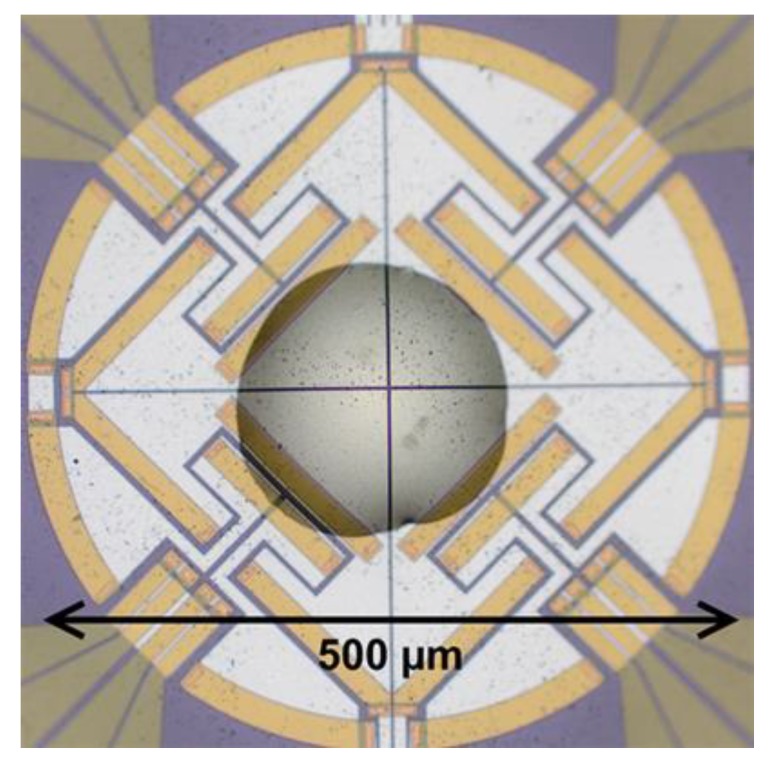
Sample of polymer on fast scanning calorimetry chip sensor.

**Figure 2:**
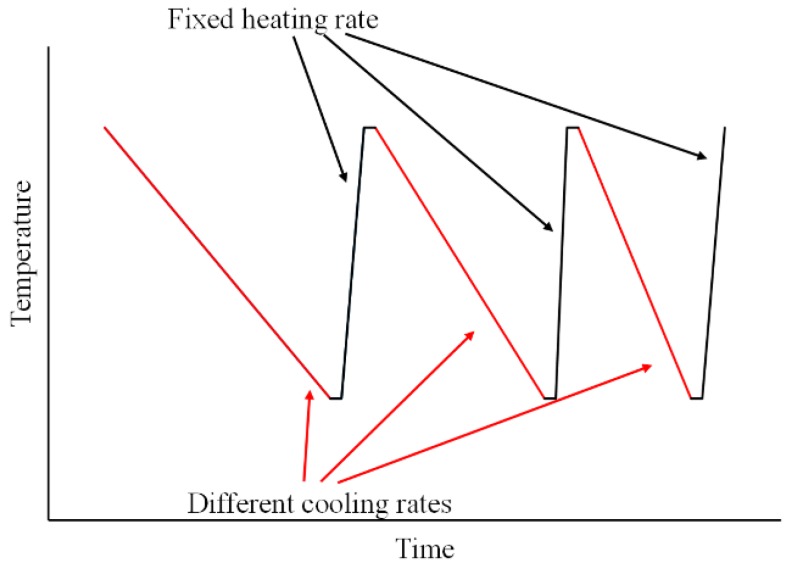
Schematic representation of the thermal program for the crystallization study of cross-linked PCL.

Fast scanning calorimetry experiments were performed using a Mettler Toledo Flash DSC 1 instrument (Mettler Toledo, Greifensee, Switzerland) with the UFS1 sensor allowing up to 300,000 K·min^−1^ (5000 K·s^−1^) heating and cooling rates. In a typical experiment, 10–50 ng of the specimen was put onto the chip sensor as shown in Figure 1, heated up to 150 °C and cooled down to −80 °C several times in order to erase the thermal memory and achieve better thermal contact with the chip sensor. After that, the specimen was repeatedly heated with the same fixed rate 300,000 K·min^−1^ and cooled with successively increasing rate from 30 to 300,000 K·min^−1^ (0.5 to 5000 K·s^−1^, see Figure 2 for a schematic representation of the thermal program).

## 3. Results

### 3.1. Equilibrium Swelling Experiments

Results of the swelling experiments for cross-linked PCL obtained from the mixtures with different percentage of BPO are given in Table 1. As expected, the cross-link density increases with increasing fraction of the radical initiator. The sample with 1% BPO has broken into small pieces during swelling, and accurate weighing necessary to determine *N* became impossible. However, these pieces could not be completely dissolved in toluene, which means that the sample is also cross-linked.

### 3.2. Conventional DSC Experiments

**Figure 3:**
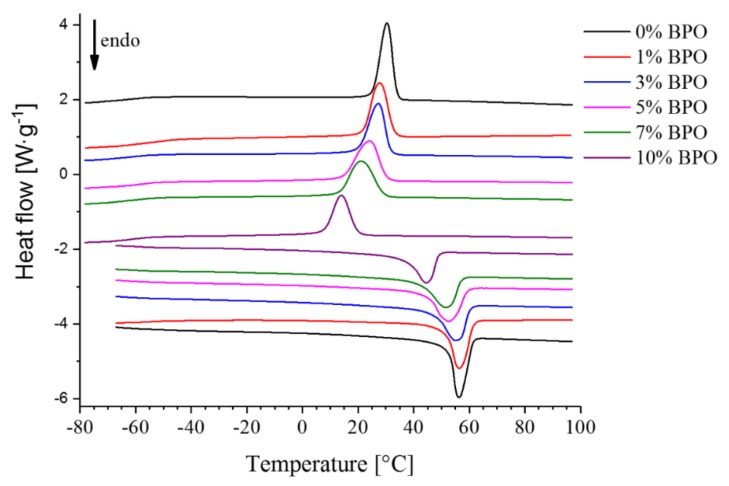
DSC cooling and subsequent heating curves recorded at 10 K·min^−1^ scanning rate.

DSC curves recorded at 10 K·min^−1^ heating rate after cooling at 10 K·min^−1^ are shown in Figure 3. The enthalpies of fusion and crystallization of the studied samples determined from these curves are also given in Table 1. They show little dependence on the cross-link density. The difference between the enthalpies of fusion and crystallization is caused by the difference in melting and crystallization temperatures and in the heat capacities of crystalline and molten states, which are about 25 K and 0.22 J·g^−1^·K^−1^ [33] for pure PCL, respectively. With these values Kirchhoff’s law provides a correction term of about 5.5 J g^−1^, explaining the value differences in Table 1. The values of the enthalpies of fusion were used to calculate the masses of the samples in FSC experiments, which cannot be done by direct weighing. According to different literature sources, 100% crystalline PCL has a melting enthalpy from 135.4 to 156.8 J g^−1^ [34], which corresponds to 47%–54% crystallinity of the unmodified PCL used for cross-linking. With increasing cross-linking density crystallinity decreases to 37%–42% for *N* = 209 mol·m^−3^. All the studied samples undergo a glass transition in the temperature range −65 to −60 °C.

Kinetics of crystallization was studied by conventional DSC experiments. Non-isothermal crystallization at constant cooling rate can be described by a modification of the Avrami model made by Jeziorny [35]. The Avrami equation

(4) $$\ln\left(1-X_{c}\right)=-Z_{t}t^{n}$$

or its linearized form

(5) $$\ln\left(-\ln\left(1-X_{c}\right)\right)=\ln Z_{t}+n\ln t$$

are commonly used for quantitative description of isothermal crystallization kinetics. Here *X*_c_ is the degree of crystallinity changing in time during the crystallization process, *n* is the Avrami exponent dependent on the mechanism of nucleation, and *Z_t_* is the crystallization rate constant. For non-isothermal crystallization, the quantity

(6) $$\ln Z_{c}=\ln Z_{t}/v$$

where *v* is the scanning rate, is supposed to be constant for fast crystallizing polymers at least at moderate scanning rates (5–20 K·min^−1^) typical for conventional DSC experiments. $Z_{c}$ is considered to be a measure of the crystallization rate of a polymer. Another quantity that allows rapid estimation of crystallization rate is crystallization half-time *t*_1/2_. It is the interval of time in which the sample cools from crystallization onset temperature to the temperature at which half of the possible crystallinity is achieved. Crystallization *t*_1/2_ decreases with increasing scanning rate. Thus, it should be compared at the same cooling rate for different polymers. In Table 2, a comparison of the values of *n* and $Z_{c}$ obtained from Avrami plots (see supplementary material for the plots), and *t*_1/2_ at 10 and 20 K·min^−1^ cooling rates is given. In all cases, the values of *n* equal 2 or slightly above. $Z_{c}$ has similar values for both cooling rates for pure PCL and two samples with the lowest cross-link densities. At 10 K·min^−1^, $Z_{c}$ decreases and *t*_1/2_ increases with increasing cross-link density *N* for all the samples except one with the largest *N*. At 20 K·min^−1^, two samples with the largest cross-link density obtained using 7% and 10% BPO crystallize faster than the one obtained using 5% BPO. Such behavior means that the crystallization process slows down with increasing cross-link density if we consider it at the same temperature. However, the drop in crystallization temperature for 7% and 10% BPO sample at 20 K·min^−1^ cooling rate is so large (possibly because of a different nucleation mechanism) that non-isothermal crystallization takes place at much lower temperature and is likely to proceed faster due to this reason.

### 3.3. FSC Experiments

**Figure 4:**
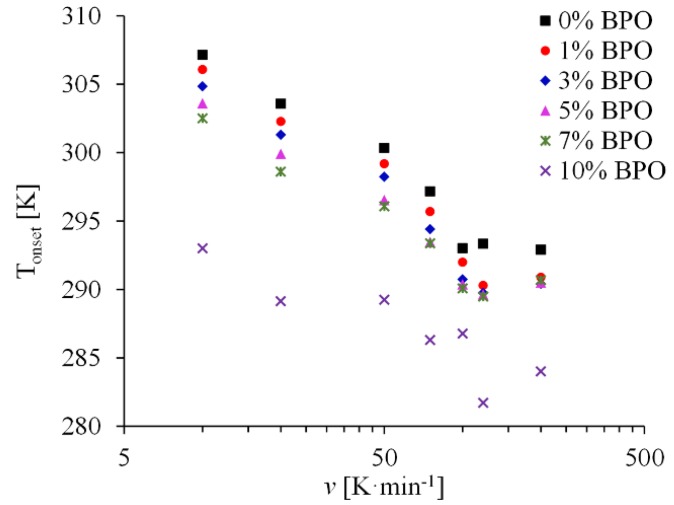
Crystallization onset temperature *T*_onset_ against the logarithm of the cooling rate for PCL and cross-linked PCL

DSC curves show that the process of crystallization of samples with higher density of cross-links starts at lower temperature (see Table 2). With increasing cooling rate, the crystallization peak position moves to lower temperatures and at some point its area starts to decrease due to incomplete crystallization during rapid cooling. The temperature corresponding to the crystallization onset changes almost linearly with the logarithm of the cooling rate (Figure 4) until it reaches certain value when the sample has not enough time to crystallize and the peak disappears. This critical cooling rate, *v*_c_, is another characteristic of crystallization kinetics. It is about 18,000 K·min^−1^ (300 K·s^−1^) [26] for unmodified PCL, which is higher than the maximum possible rate for conventional DSC instruments. FSC experiments allow to reach this and even higher cooling rates. However, it is difficult to determine the critical rate precisely from the FSC cooling curves because the crystallization peak is very small and the signal is noisy.

**Figure 5:**
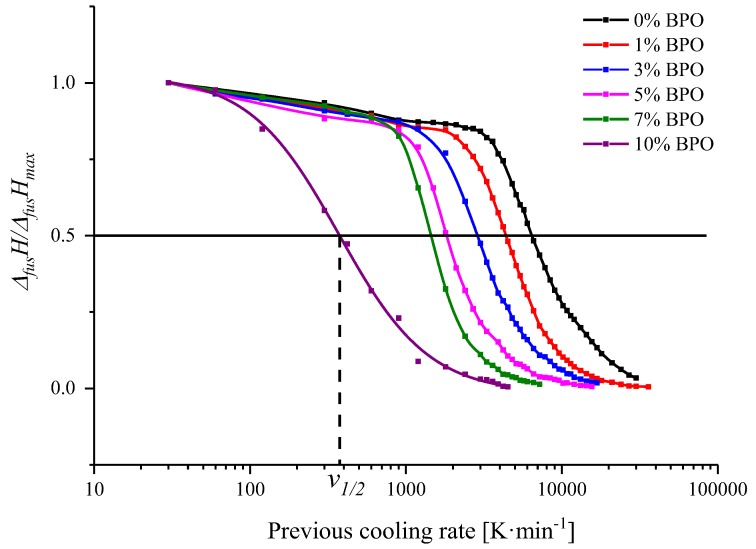
Dependence of the normalized enthalpy of melting at 300,000 K·min^−1^ (5000 K·s^−1^) heating rate on the previous cooling rate for PCL with different cross-link densities. Solid and dashed lines show the way to determine *v*_1/2_.

Therefore we made a number of scans with different cooling rates ranging from 30 to 300,000 K·min^−1^ immediately followed by reheating scans with a constant scanning rate of 300,000 K·min^−1^. Heating with this rate results in curves without the peak of cold crystallization. Thus, the melting enthalpy characterizes the total amount of crystalline phase in the polymer after cooling. The values of the melting enthalpies decrease with increasing preceding cooling rate and approach zero when the crystallization peak disappears in the cooling curves (Figure 5). Nevertheless, it is more precise and convenient to determine the cooling rate *v*_1/2_ at which the melting enthalpy reaches half of its maximum value measured at the slowest preceding cooling rate.

**Figure 6:**
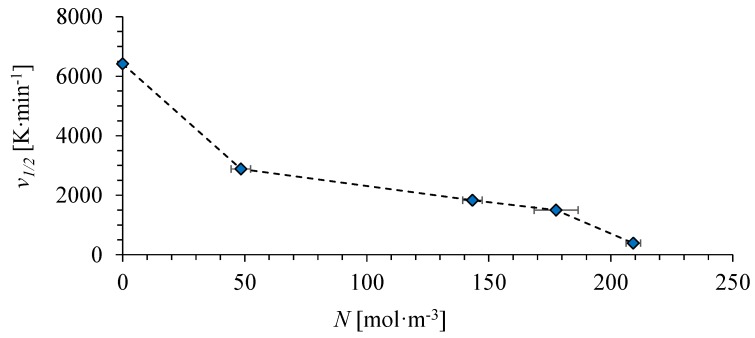
Dependence of the half-crystallization cooling rate *v*_1/2_ on the cross-link density of PCL.

The values of *v*_1/2_ are found to decrease monotonically with increasing cross-link density (Figure 6). The results were reproduced with several samples for each cross-link density. Covalent bonds between PCL chains seem to impede the crystallization process. This is similar to a conclusion made for some other cross-linked polymers from theoretical [36] and experimental [37,38,39] considerations. Such monotonic dependence makes measuring *v*_1/2_ by FSC a prospective way to characterize the degree of cross-linking of PCL with minimum sample consumption. Crystallization onset temperatures from conventional DSC requiring somewhat larger amounts of sample may also be suitable for this purpose.

## 4. Conclusions

Crystallization of cross-linked PCL was studied by means of conventional and fast scanning calorimetry. FSC clearly shows that crystallization rate decreases with increasing spatial density of cross-links. This result is in general agreement with the analysis of conventional DSC curves. However, at high crystallization temperatures the difference in crystallization rates of the samples of PCL with different cross-link densities is small, while FSC method shows quite a large change in the rates of crystallization with varying cross-link density at high supercooling. In the further studies, this difference can be a subject of more detailed analysis in isothermal crystallization experiments using FSC.

The magnitude of the cross-link density is difficult to obtain experimentally. Equilibrium swelling remains the most common method to determine it, despite its results depending on the value of the Flory parameter, which is unavailable for many polymer–solvent systems, and the Flory-Huggins theory of polymer solutions being quite a rough approximation itself. Furthermore, swelling studies require precise determination of the mass or volume of the swollen sample, which can be very imprecise and require large polymer samples. Thus, the search for alternative methods to estimate the cross-link densities remains an actual problem. A few methods based on the mechanical properties of the polymer, Raman, and NMR spectroscopy were previously suggested [29,40,41,42]. Our study shows that it is possible to use FSC for this purpose by determining the cooling rate at which half of the possible crystallinity is achieved. FSC requires a very small amount of sample that can be cut even from the final product.

## Figures and Tables

**Table 1:** Swelling ratios, cross-link densities, and enthalpies of fusion and crystallization at 10 K·min^−1^ cooling rate for the samples of PCL and cross-linked PCL obtained using various amounts of BPO.

Weight % of BPO	*Q*	*N*/mol·m^−3^	Δ_fus_*H*/J·g^−1^	Δ_cryst_*H*/J·g^−1^
0	0	0	73.5 ± 2.1	−67.6 ± 1.9
1			72.9 ± 2.6	−64.3 ± 2.4
3	14.9 ± 0.5	48.4 ± 4	71.7 ± 1.8	−65.2 ± 2.1
5	8.2 ± 0.1	143.3 ± 4	68.7 ± 2.4	−65.2 ± 1.0
7	7.4 ± 0.2	177.6 ± 9	67.5 ± 2.0	−60.6 ± 1.7
10	6.7 ± 0.04	209.2 ± 3	57.3 ± 1.2	−49.2 ± 1.8

**Table 2:** Crystallization onset temperatures, half-times, and kinetic parameters of non-isothermal crystallization determined using the modified Avrami method.

Weight % of BPO	Cooling Rate/K·min^−1^	*T*_onset_/K	*t*_1/2_/s	*n*	Z_c_/min^−*n*^·K^−1^
0	10	307.2	27	2.05	1.13
0	20	303.6	13	1.96	1.14
1	10	306.1	33	2.04	1.09
1	20	302.3	22	1.96	1.08
3	10	304.9	33	2.01	1.09
3	20	301.3	22	1.96	1.08
5	10	303.6	45	2.12	1.02
5	20	299.9	29	2.01	1.06
7	10	302.5	50	2.39	1.01
7	20	298.6	29	2.33	1.07
10	10	293.0	37	2.24	1.07
10	20	289.2	23	2.21	1.09

## Supplementary Materials

The supplementary material containing the Avrami model plots is available online at http://www.mdpi.com/2073-4360/10/8/902/s1.
