# Supplementary material for: Influence of the Cross-Link Density on the Rate of Crystallization of Poly(ε-Caprolactone)
Source: Polymers (Basel). 2018 Aug 11;10(8):902. doi: 10.3390/polym10080902 (PMC6404166; doi:10.3390/polym10080902)
Supplement: Supplementary file 1 [file polymers-10-00902-s001.pdf]

Supplementary material for "Influence of the cross-link density on the rate of crystallization of poly(ε-caprolactone)"

Avrami model plots of conventional DSC data

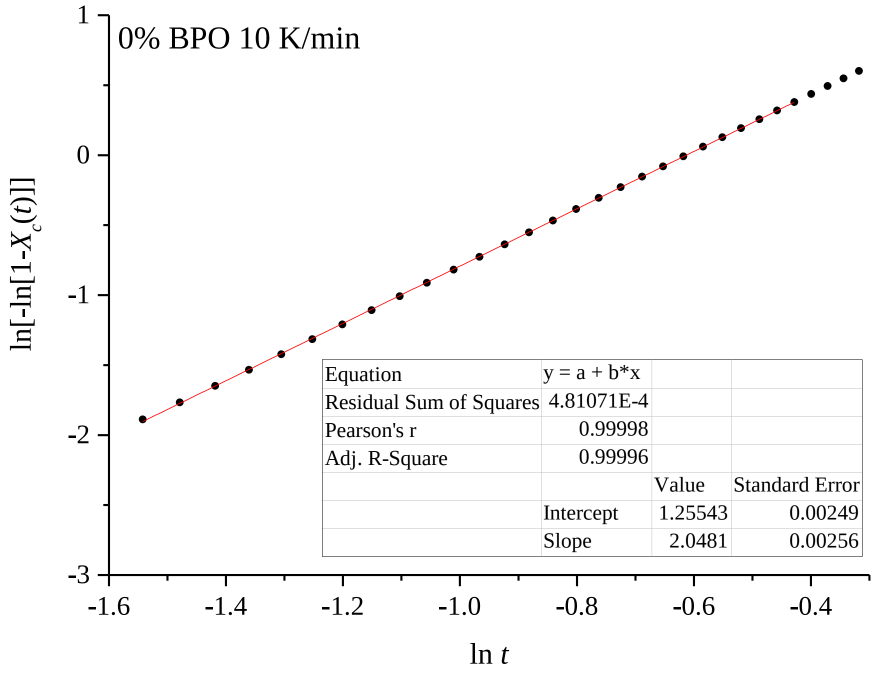

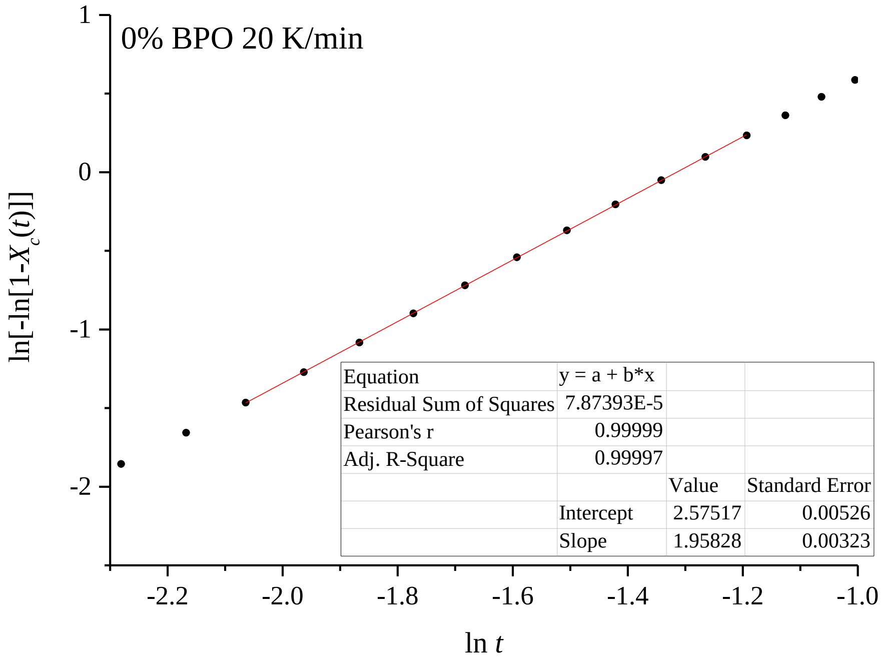

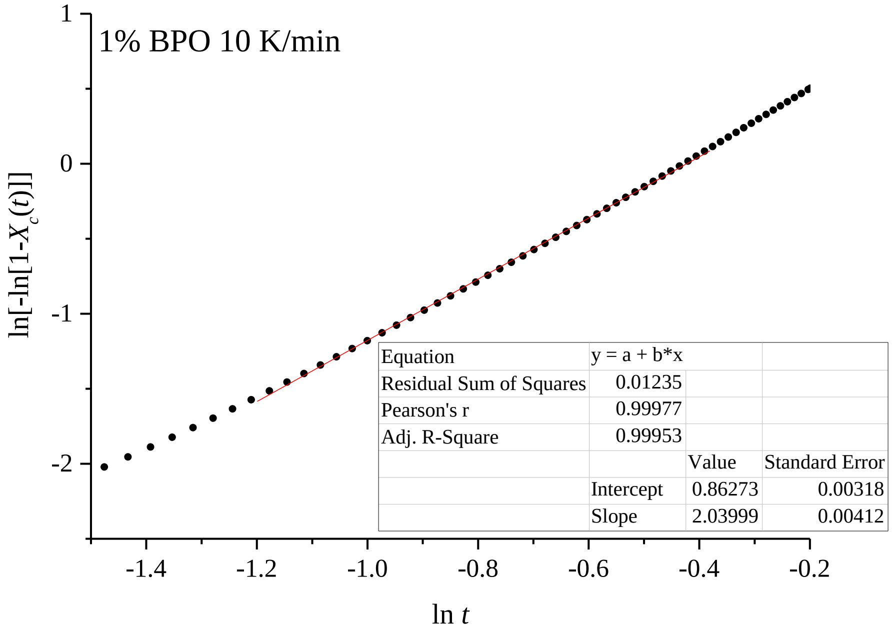
1% BPO 10 K/min
ln[-ln[1-$X_c(t)$]]
ln t
1
0
-1
-2
-1.4
-1.2
-1.0
-0.8
-0.6
-0.4
-0.2
Equation
y = a + b*x
Residual Sum of Squares
0.01235
Pearson's r
0.99977
Adj. R-Square
0.99953
Value
Standard Error
Intercept
0.86273
0.00318
Slope
2.03999
0.00412

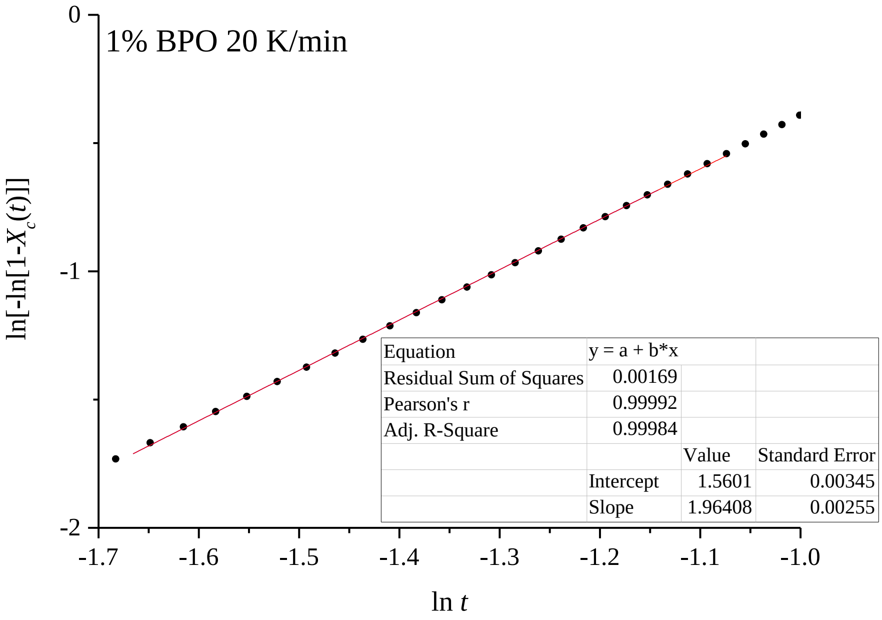
1% BPO 20 K/min
ln[-ln[1-$X_c(t)$]]
ln t
0
-1
-2
-1.7
-1.6
-1.5
-1.4
-1.3
-1.2
-1.1
-1.0
Equation
y = a + b*x
Residual Sum of Squares
0.00169
Pearson's r
0.99992
Adj. R-Square
0.99984
Value
Standard Error
Intercept
1.5601
0.00345
Slope
1.96408
0.00255

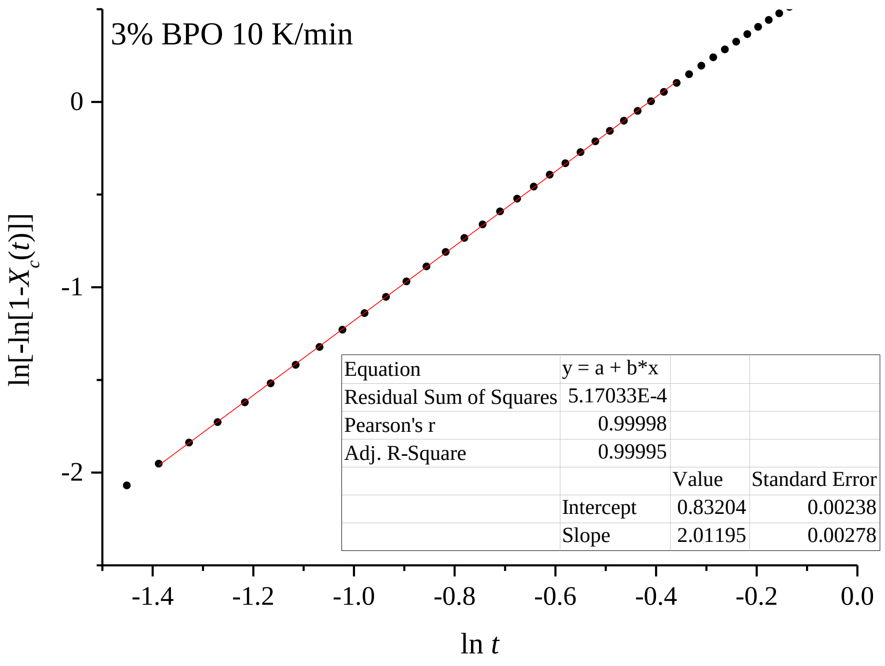
3% BPO 10 K/min
ln[-ln[1-X_c(t)]]
ln t
0
-1
-2
-1.4
-1.2
-1.0
-0.8
-0.6
-0.4
-0.2
0.0
Equation
y = a + b*x
Residual Sum of Squares
5.17033E-4
Pearson's r
0.99998
Adj. R-Square
0.99995
Value
Standard Error
Intercept
0.83204
0.00238
Slope
2.01195
0.00278

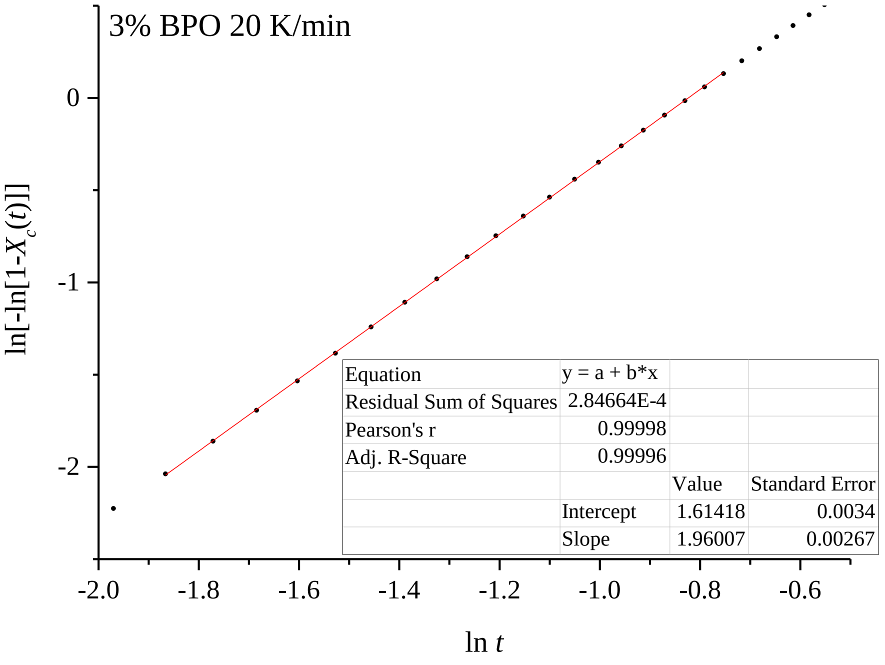
3% BPO 20 K/min
ln[-ln[1-X_c(t)]]
ln t
0
-1
-2
-2.0
-1.8
-1.6
-1.4
-1.2
-1.0
-0.8
-0.6
Equation
y = a + b*x
Residual Sum of Squares
2.84664E-4
Pearson's r
0.99998
Adj. R-Square
0.99996
Value
Standard Error
Intercept
1.61418
0.0034
Slope
1.96007
0.00267

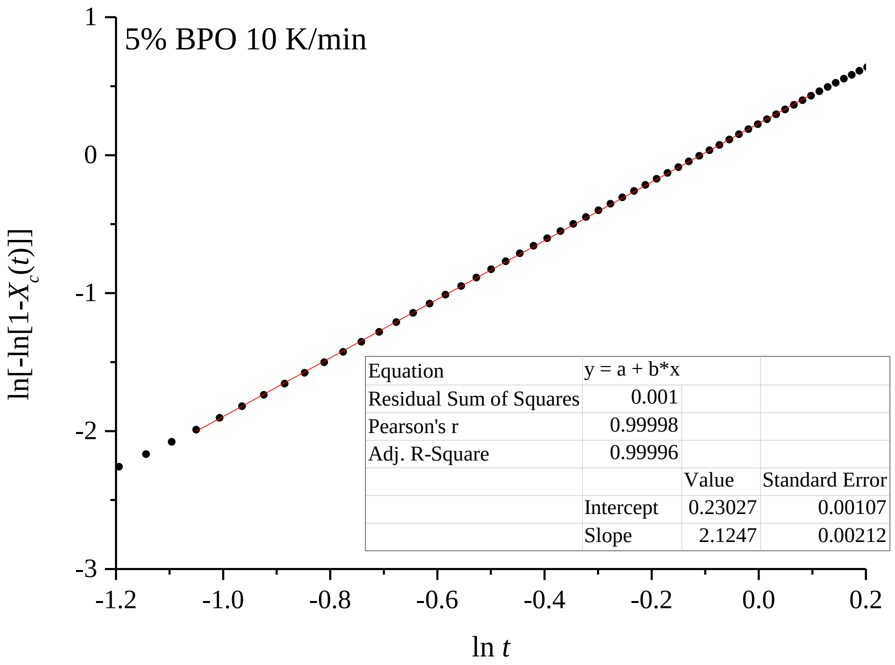
5% BPO 10 K/min
ln[-ln[1-X_c(t)]]
ln t
1
0
-1
-2
-3
-1.2
-1.0
-0.8
-0.6
-0.4
-0.2
0.0
0.2
Equation
y = a + b*x
Residual Sum of Squares
0.001
Pearson's r
0.99998
Adj. R-Square
0.99996
Value
Standard Error
Intercept
0.23027
0.00107
Slope
2.1247
0.00212

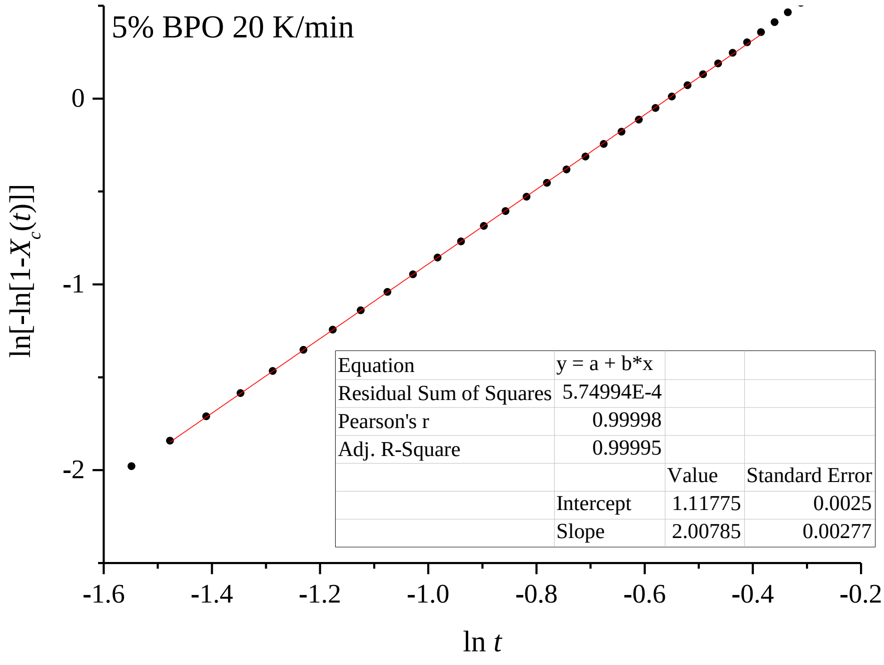
5% BPO 20 K/min
ln[-ln[1-X_c(t)]]
ln t
0
-1
-2
-1.6
-1.4
-1.2
-1.0
-0.8
-0.6
-0.4
-0.2
Equation
y = a + b*x
Residual Sum of Squares
5.74994E-4
Pearson's r
0.99998
Adj. R-Square
0.99995
Value
Standard Error
Intercept
1.11775
0.0025
Slope
2.00785
0.00277

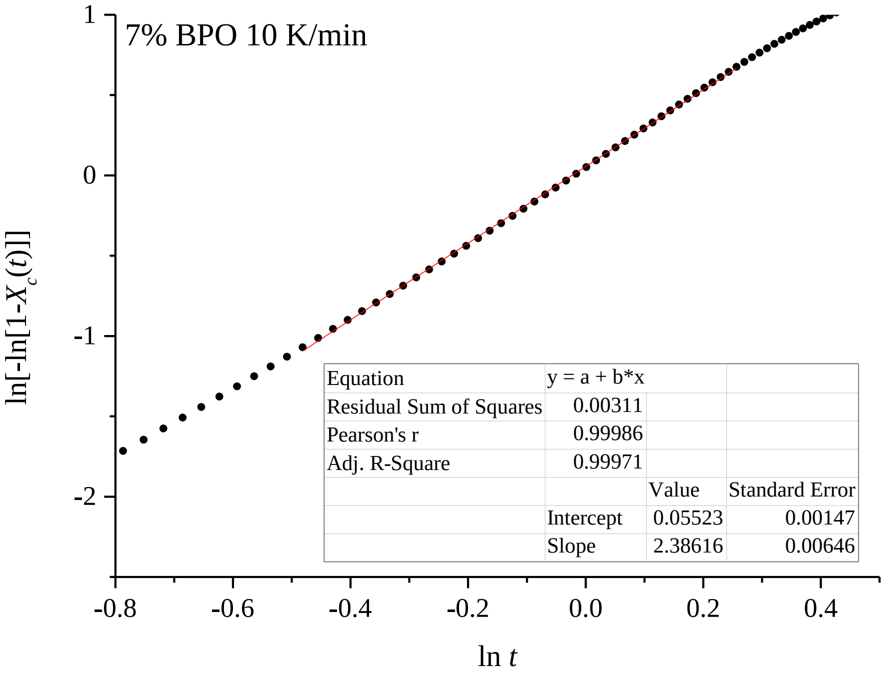
7% BPO 10 K/min
ln[-ln[1-$X_c(t)$]]
ln t
1
0
-1
-2
-0.8
-0.6
-0.4
-0.2
0.0
0.2
0.4
Equation
y = a + b*x
Residual Sum of Squares
0.00311
Pearson's r
0.99986
Adj. R-Square
0.99971
Value
Standard Error
Intercept
0.05523
0.00147
Slope
2.38616
0.00646

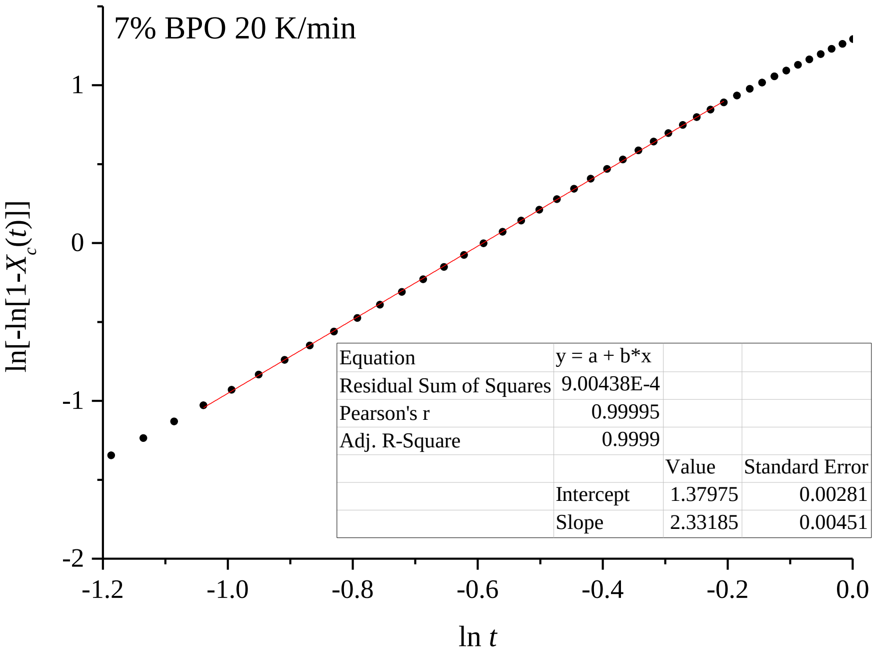
7% BPO 20 K/min
ln[-ln[1-$X_c(t)$]]
ln t
1
0
-1
-2
-1.2
-1.0
-0.8
-0.6
-0.4
-0.2
0.0
Equation
y = a + b*x
Residual Sum of Squares
9.00438E-4
Pearson's r
0.99995
Adj. R-Square
0.9999
Value
Standard Error
Intercept
1.37975
0.00281
Slope
2.33185
0.00451

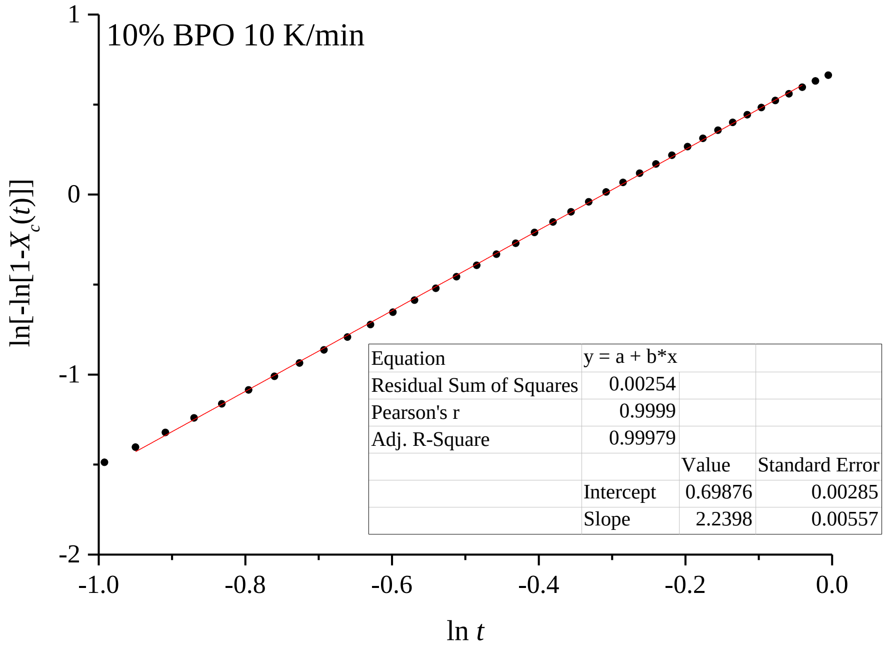
10% BPO 10 K/min
ln[-ln[1-X_c(t)]]
ln t
1
0
-1
-2
-1.0
-0.8
-0.6
-0.4
-0.2
0.0
Equation y = a + b*x
Residual Sum of Squares 0.00254
Pearson's r 0.9999
Adj. R-Square 0.99979
Value Standard Error
Intercept 0.69876 0.00285
Slope 2.2398 0.00557

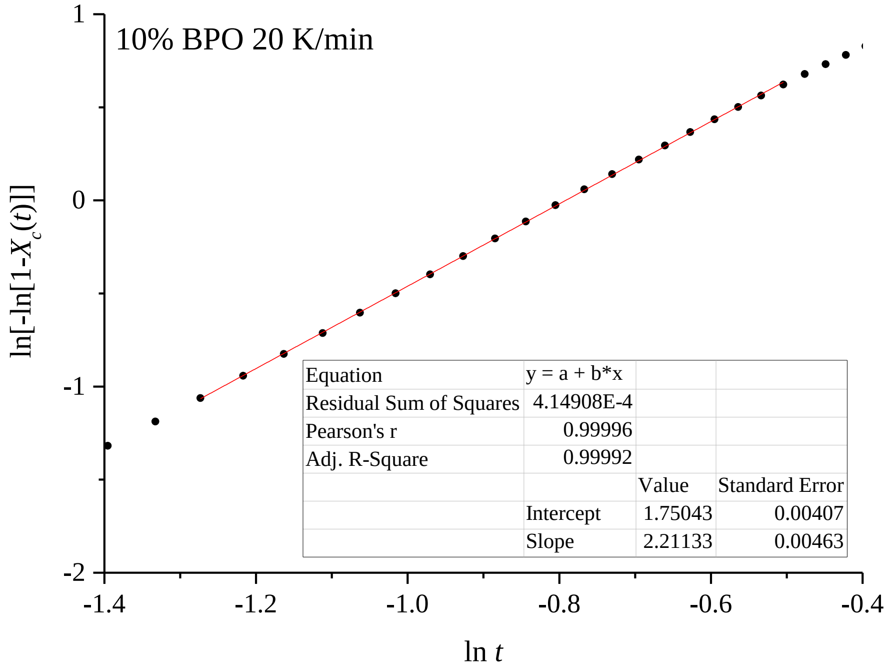
10% BPO 20 K/min
ln[-ln[1-X_c(t)]]
ln t
1
0
-1
-2
-1.4
-1.2
-1.0
-0.8
-0.6
-0.4
Equation y = a + b*x
Residual Sum of Squares 4.14908E-4
Pearson's r 0.99996
Adj. R-Square 0.99992
Value Standard Error
Intercept 1.75043 0.00407
Slope 2.21133 0.00463
